# A new class of biological ion-driven rotary molecular motors with 5:2 symmetry

**DOI:** 10.3389/fmicb.2022.948383

**Published:** 2022-08-05

**Authors:** Martin Rieu, Roscislaw Krutyholowa, Nicholas M. I. Taylor, Richard M. Berry

**Affiliations:** ^1^Department of Physics, University of Oxford, Oxford, United Kingdom; ^2^Kavli Institute for Nanoscience Discovery, Dorothy Crowfoot Hodgkin Building University of Oxford, Oxford, United Kingdom; ^3^Department of Biology, Institute of Molecular Biology and Biophysics, ETH Zurich, Zurich, Switzerland; ^4^Novo Nordisk Foundation Center for Protein Research, University of Copenhagen, Copenhagen, Denmark

**Keywords:** bacterial flagellar motility, molecular machine, cryo EM, structure function, rotary motor

## Abstract

Several new structures of three types of protein complexes, obtained by cryo-electron microscopy (cryo-EM) and published between 2019 and 2021, identify a new family of natural molecular wheels, the “5:2 rotary motors.” These span the cytoplasmic membranes of bacteria, and their rotation is driven by ion flow into the cell. They consist of a pentameric wheel encircling a dimeric axle within the cytoplasmic membrane of both Gram-positive and gram-negative bacteria. The axles extend into the periplasm, and the wheels extend into the cytoplasm. Rotation of these wheels has never been observed directly; it is inferred from the symmetry of the complexes and from the roles they play within the larger systems that they are known to power. In particular, the new structure of the stator complex of the Bacterial Flagellar Motor, MotA_5_B_2_, is consistent with a “wheels within wheels” model of the motor. Other 5:2 rotary motors are believed to share the core rotary function and mechanism, driven by ion-motive force at the cytoplasmic membrane. Their structures diverge in their periplasmic and cytoplasmic parts, reflecting the variety of roles that they perform. This review focuses on the structures of 5:2 rotary motors and their proposed mechanisms and functions. We also discuss molecular rotation in general and its relation to the rotational symmetry of molecular complexes.

## Introduction

Nature has made very little use of the wheel for locomotion. On the macroscopic scale, there are good reasons for this. First, there is the problem of how to exchange nutrients and other metabolic necessities with a wheel that needs to be physically separate from the rest of the organism if it is to rotate. Furthermore, without flat roads, a wheel is not very effective for animal locomotion. Neither of these problems exists, however, for life on the micro- and nano-scales, where diffusion solves the nutrient transport problem and immersion in aqueous solutions makes “roads” irrelevant. The first-discovered natural wheel is the Bacterial Flagellar Motor (BFM), a rotary device that propels swimming bacteria (Wadhwa and Berg, 2021). Rotation of the BFM was proposed in 1973 (Berg and Anderson, 1973), and much has been learned about it since then. Until 2020, the only other known wheels in nature were the family of F-, V- and A-type ATPases (Müller and Grüber, 2003; Kühlbrandt and Davies, 2016; Guo and Rubinstein, 2018). Rotation of the F_1_-ATPase coupled to ATP hydrolysis was inferred from biochemical evidence (Duncan and Bulygin, 1995) and later observed directly in 1997 (Noji et al., 1997), and the rotational mechanism has been described in considerable detail (Noji et al., 1997; Sobti et al., 2021; Guo and Rubinstein, 2022; Noji and Ueno, 2022). In its native setting, the F_1_ component of the F_1_F_O_ ATP-synthase synthesizes ATP as F_O_ turns in response to the ion-motive force. In the reverse reaction, ATP hydrolysis by F_1_ can drive the rotation of F_O_ in the opposite direction, leading to the pumping of ions out across the membrane. Rotation is also a component of the motion of other known molecular machines, including many that move relative to helical DNA molecules, usually coupled to ATP hydrolysis. But this class of motion is closer to a nut and bolt or screw than to a wheel.

Several new structures of three types of protein complexes, obtained by cryo-electron microscopy (cryo-EM) and published between 2019 and 2021, identify a new family of natural molecular wheels, the “5:2 rotary motors.” These consist of a pentameric wheel encircling a dimeric axle within the cytoplasmic membrane of both Gram-positive and Gram-negative bacteria. The axles extend into the periplasm, and the wheels extend into the cytoplasm. Rotation of these wheels has never been observed directly; it is inferred from the symmetry of the complexes and from the roles they play within the larger systems that they are known to power. In particular, the new structure of the stator complex of the BFM, MotA_5_B_2_, is consistent with a “wheels within wheels” model of the BFM. To quote the late Howard Berg: “So, remarkably, the rotary flagellar motor is powered by an array of even smaller rotary motors. This represents a major shift in thinking about how the stator units operate” (Wadhwa and Berg, 2021). Other 5:2 rotary motors are believed to share the core rotary function and mechanism, driven by ion-motive force at the cytoplasmic membrane. Their structures diverge in their periplasmic and cytoplasmic parts, reflecting the variety of roles that they perform. This review focuses on general aspects of the structures of 5:2 rotary motors and their proposed mechanisms and functions.

We also discuss molecular rotation in general and its relation to the rotational symmetry of molecular complexes. Rotation is a natural way to achieve a repetitive process within a confined space. It is also an effective way to generate locomotion, similar to either a wheeled vehicle or the propellers of boats and aeroplanes. Any rotary machine is defined by the parts that rotate relative to each other. By convention, the part that is anchored to its surroundings is called the “stator,” and the other is called the “rotor.”

Because equivalent components in different 5:2 motors may be anchored in some cases and not in others, and because the entire MotA_5_B_2_ complex has long been identified as the BFM “stator unit,” we will avoid using the terms “stator” and “rotor” for the parts of 5:2 motors and rotary complexes, reserving them for parts of the entire BFM. Instead, to avoid confusion, we will use “outer” to designate the part further from the rotation axis and “inner” for the other. Known rotary molecular motors can be characterized by symmetry mismatches between the inner and outer parts. These mismatches are thought to be intrinsic to their rotary mechanism. For example, the outer:inner symmetry ratio for F_1_ is 3:1, and for F_O_ it is 1:n, where the c-ring rotational symmetry n ranges from 8 to 15 (Cheuk and Meier, 2021). Rotational symmetries in the BFM are large and variable: current rotor structures are 34- and 46-fold (other rotor symmetries may be discovered in future), and the number of stator units can vary between 1-fold and 18-fold (Chang et al., 2021). However, the discovery that the repeating unit of the BFM stator is a 5:2 rotary motor, which is now believed to be entirely responsible for torque generation, indicates that BFM symmetry mismatches are not intrinsic to its fundamental mechanism. The discovery of the family of 5:2 rotary motors raises the question of whether rotary motors with other symmetries remain to be discovered, and what considerations select for which symmetries. We address these and other symmetry-related questions in later parts of this article.

## Structures of ion-driven 5:2 rotary motors

### MotAB

**Figure 1:**
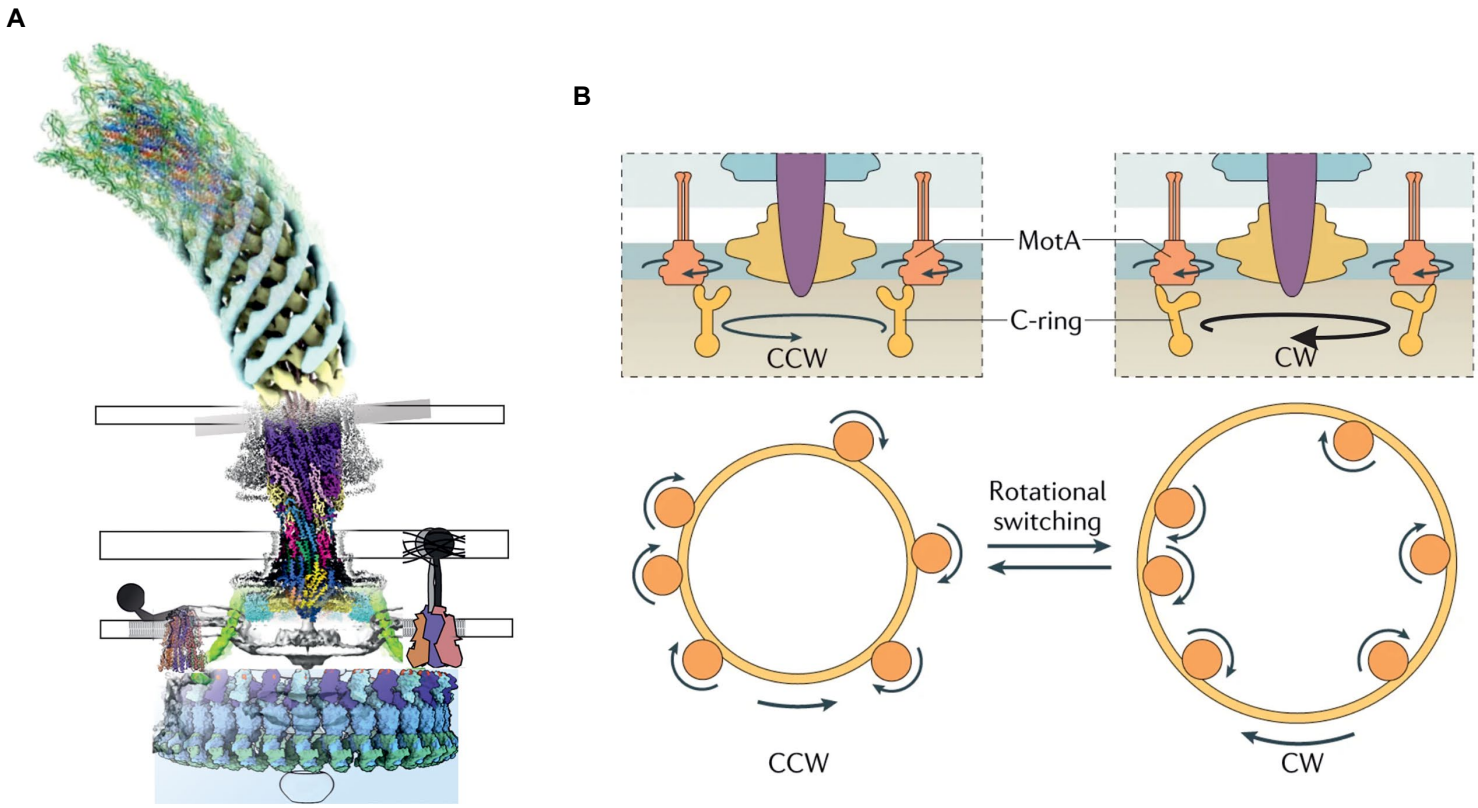
**(A)** A composite model of the bacterial flagellar motor based on the published structures. **(B)** The current model of rotation and switching of the BFM driven by the unidirectional rotation of MotAB. (**B** adapted from: https://www.nature.com/articles/s41579-021-00626-4).

Many species of bacteria utilize flagella to move. Flagella generate propulsion *via* clockwise (CW) or counterclockwise (CCW) rotation (as viewed, per convention, from outside the cell) of a helical extracellular filament stemming from the hook, which is attached to the BFM rotor (Armitage and Berry, 2020). The BFM is a circular transmembrane macromolecular assembly approximately 60 nm in diameter, depending on the species (Chang et al., 2020). In Gram-negative bacteria, it consists of obligatory L-, P-, MS- and C-ring structures that owe their names to their positions relative to the membrane (Zhu et al., 2017). Some species have additional, often large, stator structures (reviewed by Terashima et al., 2017), for example, the H- and T-rings that are hypothesized to be necessary in sodium-driven BFMs (Merino and Tomás, 2016; Zhu et al., 2017). In *Escherichia coli* and *Salmonella enterica*, rotation of the C-ring in either direction is driven by multiple independent MotA_5_B_2_ stator units, whose rotation is driven by H^+^ influx across the cytoplasmic membrane, which is driven by the protonmotive force (PMF). In other species other ions replace H^+^; for example, *Vibrio alginolyticus* has Na^+^-driven stator units called PomA_5_B_2_. Stator units are now believed to rotate unidirectionally, pushing a ring of FliG on the periphery of the C-ring *via* either their proximal or distal parts (relative to the rotation axis of the entire BFM) to drive CCW or CW rotation, respectively (Carroll et al., 2020). The overall structure and mechanism of the BFM are discussed further below and illustrated in Figure 1. The MS- and C-rings near the cytoplasmic membrane are parts of the rotor and are present in all BFMs, whereas the L- and P- rings are stationary bushings that transmit rotation through the outer membrane and are unique to Gram-negative bacteria.

**Figure 2:**
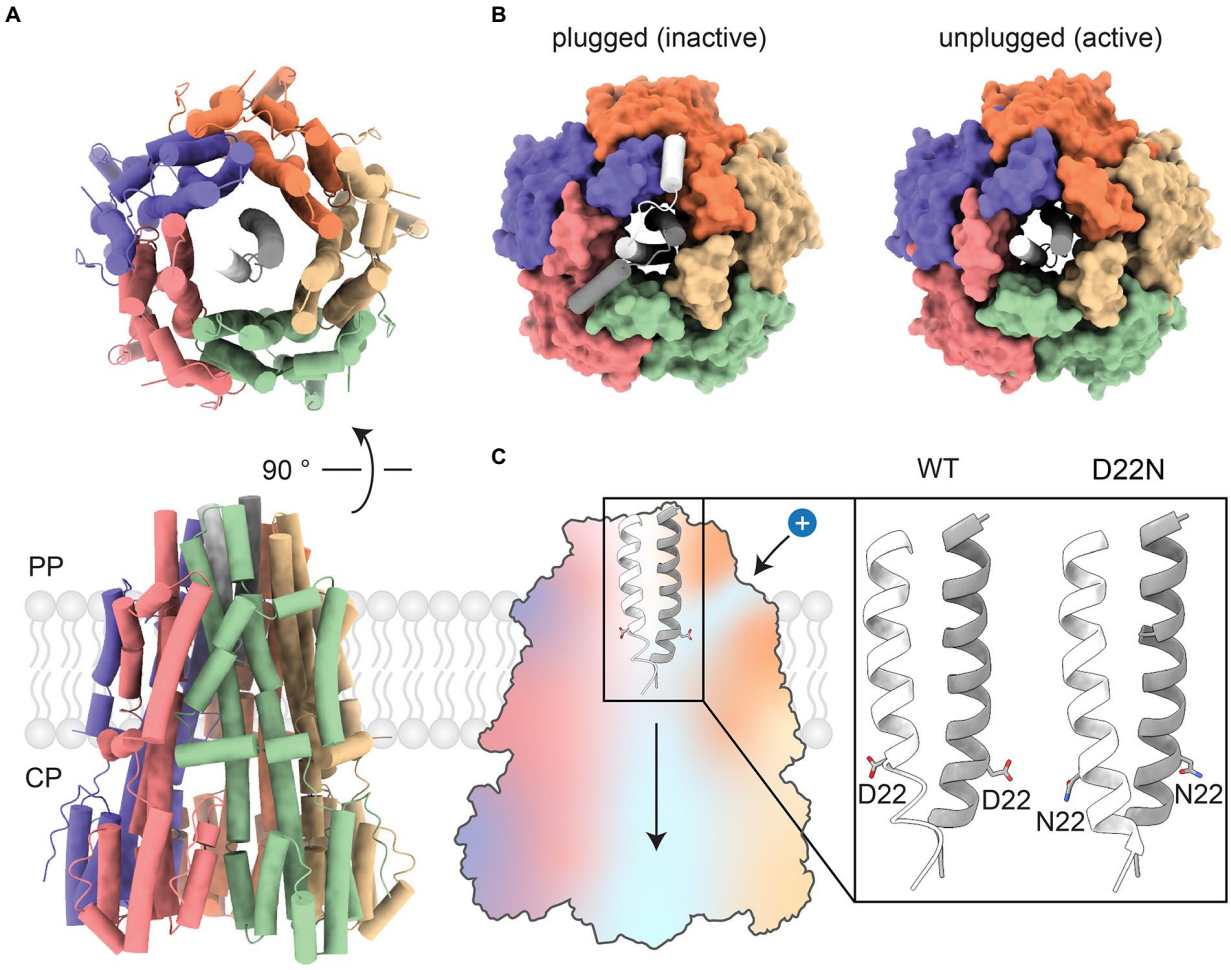
Overview of the *Cj*MotA/MotB complex. **(A)** Top and front views of the *Cj*MotA (colored)/*Cj*MotB (white and gray) protein complex in 5:2 stoichiometry (PDB ID:6YKP). **(B)** Comparison of plugged (left, inactive, PDB ID: 6YKM) and unplugged (right, active, PDB ID: 6YKP) complexes. Plugs mechanically block the rotation of the MotA pentamer relative to MotB. **(C)** (Left) Proton channels for proton influx and dissociation in MotA/B complex are shown as blue areas on the cross-section of the MotA/B complex. (Right) Close-up view of MotB helices with D22 highlighted. Unprotonated side chains of aspartic acid (D22) point upward, facing the proton inlet channel (PDB ID: 6YKP). The protonation-mimetic residue substitution D22N reorients the sidechain of residue 22 downward, enabling MotA/B movement (PDB ID: 6YKR).

BFM stator units are molecular assemblies of approximately 200 kDa encoded by the products of two genes from a single operon (Liu and Ochman, 2007). Each unit comprises a single “5:2 rotary motor,” in which an outer part consisting of five transmembrane, predominantly alpha-helical, MotA subunits surrounds a cavity that accommodates an inner part consisting of the two N-terminal transmembrane helices of a MotB dimer (Figure 2A). Within the MotA protein, two domains can be clearly distinguished – a transmembrane domain, involved in proton-driven torque generation, and a cytoplasmic domain, which transfers mechanical energy to FliG in the C-ring. The crystal structure of the C-terminal domain of MotB revealed a peptidoglycan-binding domain that anchors the MotAB stator unit to the cell wall and restricts lateral in-membrane diffusion, keeping MotAB available for the C-ring. Several studies shed light on the structures of periplasmic domains of MotB from *Helicobacter pylori*, *S. enterica*, and PomB from *V. alginolyticus* (Kojima et al., 2009; Oneill et al., 2011; Zhu et al., 2014). The N-termini of the MotB dimer comprises an axle for MotA rotation, followed by a short helical plug that mechanically fixes the position of MotA relative to MotB, stopping rotation (Morimoto et al., 2010; Homma et al., 2021; Figure 2B). Genetic deletion of the plug reduces bacterial growth rates (Hosking et al., 2006) because the uncontrolled proton flow acidifies the cytoplasm.

It is likely that the periplasmic side of MotA contains narrow H^+^ channels that allow protons to flow from the periplasm to a binding site at a conserved aspartate residue (D22 in *Campylobacter jejuni*, D32 in *E. coli* (Zhou et al., 1998) and D33 in *S. enterica*) mid-way across the membrane on MotB (Figure 2C). Proton binding neutralizes the otherwise negatively charged D22. This is mimicked by the mutation MotB D22N, which induces only very limited changes (in a structure from *C. jejuni*) – mainly a reorientation of one of the side chains of residue 22 when the structure of the unplugged wild-type stator is compared to the structure of the unplugged D22N stator. These observations, along with the 5:2 rotational symmetry, suggest a model for the mechanism of coupling proton flux to the rotation of MotAB and the other 5:2 rotary motors, which is described below.

**Figure 3:**
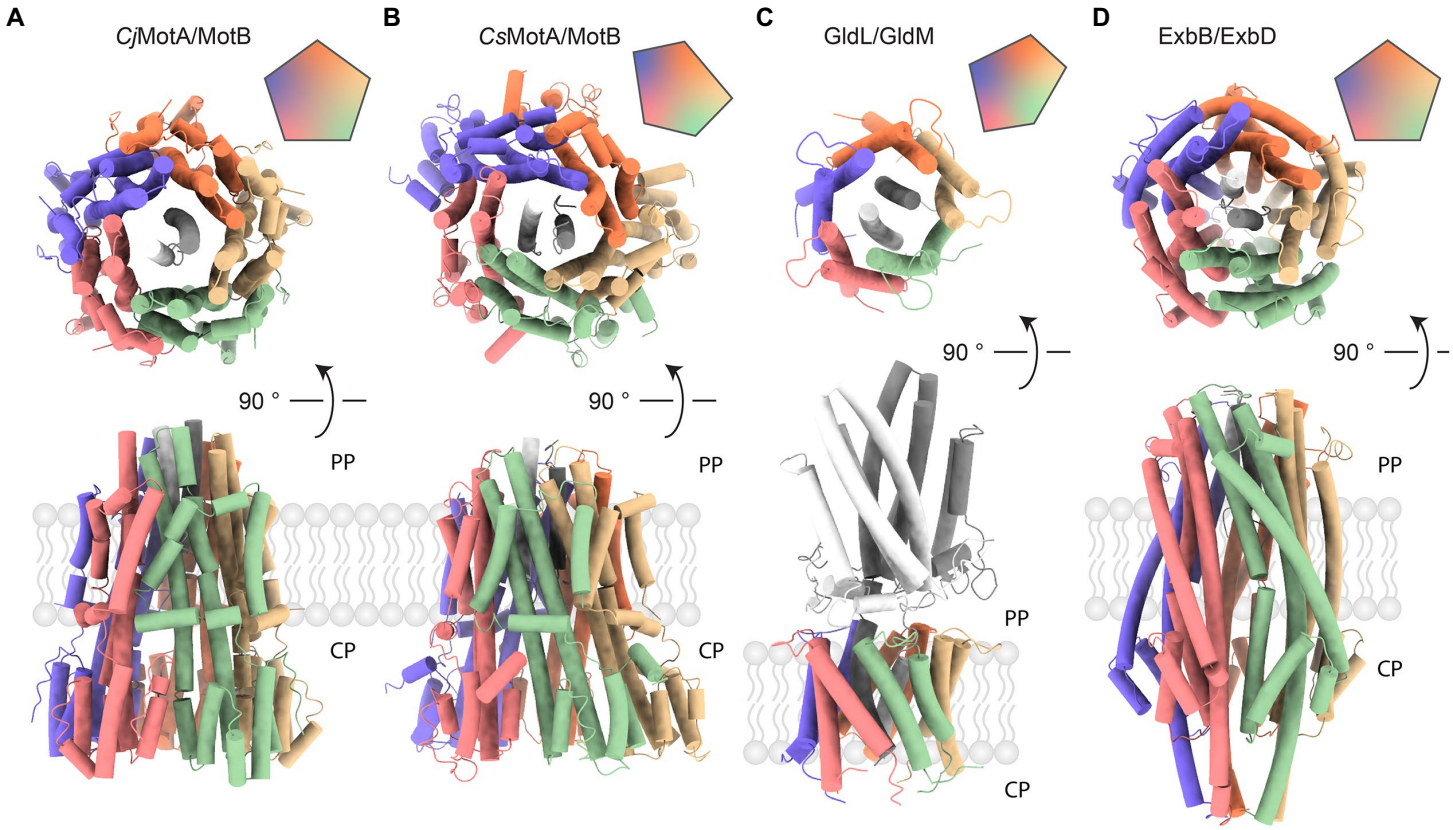
Comparison of complexes with 5:2 stoichiometry. Vertical (top) and horizontal (bottom) views of protein complexes with 5:2 stoichiometry. Assembly symmetry is represented by the top right insert. PP stands for periplasm, CP indicates cytoplasm. **(A)** MotA/B complex from a Gram-negative *Campylobacter jejuni* (PDB ID: 6YKP). **(B)** MotA/B complex from a Gram-positive *Clostridium sporogenes* (PDB ID: 6YSF). **(C)** GldL/M complex from a Gram-negative *Flavobacterium johnsoniae* (PDB ID: 6YS8). The periplasmic domain is shown in gray. **(D)** ExbB/D complex from a Gram-negative *Serratia marcescens* (PDB ID: 7AJQ). ExbB (colored) accommodates two helices from ExbD (shades of gray) that serve as a rotational axis.

Recently, cryo-EM-based atomic models have been built for the H^+^-dependent stator units of *C. jejuni*, *Bacillus subtilis*, and *Clostridium sporogenes* (Deme et al., 2020; Santiveri et al., 2020). EM maps have also been obtained for the Na^+^ −driven *V. alginolyticus* and *Vibrio mimicus* PomAB and the H^+^-driven *Shewanella oneidensis* MotAB. All of them share the same 5:2 stoichiometry and have a common subunit arrangement. However, there are some noticeable differences. For instance, the MotAB stator units from Gram-negative bacteria possess a slightly bigger periplasmic/extracellular part than their Gram-positive counterparts (Figures 3A,B). In addition, the MotA pentamer from Gram-negative *C. jejuni* organizes with near-perfect C5 symmetry, whereas the C5 symmetry of the MotA pentamer from a Gram-positive *C*. *sporogenes* is more distorted. The potential mechanistic causes of asymmetry in the case of *C. sporogenes* MotA have been discussed (Deme et al., 2020). However, it is not clear whether such a difference is systematic between Gram-positive and Gram-negative bacteria or whether it is observed due to enhanced stability of alternative or intermediate states captured by cryo-EM in particular species. The difference between a symmetric structure of *B*. *subtilis* MotAB and an asymmetric *C. sporogenes* MotAB, both Gram-positive, favor the stability-related hypothesis. Additional structures of Gram-positive and Gram-negative stators should resolve this issue.

### ExbBD

**Figure 4:**
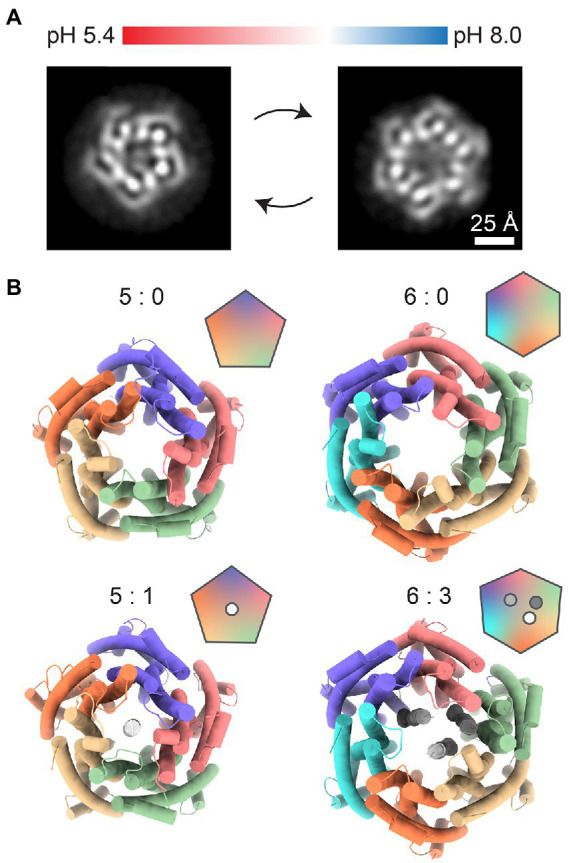
Other possible stoichiometries of the ExbB/D complex. **(A)** Changes in pH shift the equilibrium between pentameric (left) and hexameric (right) ExbB/D complexes. Images represent relevant 2D class averages from Maki-Yonekura et al. (2018). **(B)** Top views of previously solved ExbB/D complexes. Labels indicate the ExbB:ExbD ratio. The 5:0 complex (PDB ID: 5SV0) was published by Celia et al. (2016). The 5:1 (PDB ID: 5ZFV), 6:0 (PDB ID: 5ZFP) and 6:3 (PDB ID: 5ZFU) complexes were published by Maki-Yonekura et al. (2018).

Another example of a structurally characterized 5:2 rotary motor is the ExbBD complex, which is vital for the function of the Ton system responsible for the uptake of iron ions, vitamin B12, and biopolymers (Faraldo-Gómez and Sansom, 2003; Noinaj et al., 2010; Krewulak and Vogel, 2011; Celia et al., 2020). In Gram-negative bacteria, the ExbBD complex is located in the inner membrane (IM). It utilizes the PMF to activate transport at TonB-dependent receptors, over ~20 nm distant in the outer membrane (OM) (Du et al., 2014; Celia et al., 2020; Ratliff et al., 2021). The C-terminal domain of TonB interacts with TonB-dependent receptors and induces conformational changes which promote ligand uptake. Recent cryo-EM reconstructions of ExbBD from *E. coli* and *Serratia marcescens* reveal the familiar 5:2 arrangement (Figure 3D; Celia et al., 2019; Biou et al., 2021). As in MotAB, five transmembrane ExbB proteins surround two central ExbD helices. Unfortunately, existing cryo-EM reconstructions lack structural information on other parts of the complex that are important for its function: a relatively large cytoplasmic domain of ExbB and the periplasmic domain of ExbD. Other important parts of the TonB-ExbBD structure remain to be solved (Maki-Yonekura et al., 2018). Despite the fact that the C-terminal domain of TonB bound to several TonB-dependent receptors has been structurally characterized, the question of specificity and regulation of these interactions also remains largely unanswered (Pawelek et al., 2006; Josts et al., 2019). Cryo-EM studies from 2016 and 2018 observed several different stoichiometries of *E. coli* ExbB:D, which varied from 5:0, 5:1, 6:0, and 6:3, depending on pH and purification protocol (Figures 4A,B; Celia et al., 2016; Maki-Yonekura et al., 2018). It remains to be seen whether these forms have any physiological relevance or are artifacts of sample preparation.

### GldLM

Gram-negative Bacteroidetes utilize another 5:2 motor, the GldLM complex, as a proton-driven engine for gliding motility on solid surfaces and to power type IX secretion systems (McBride, 2019; Wadhwa and Berg, 2021). A structure of the *Flavobacterium johnsoniae* GldLM complex (homologous to *Porphryomonas gingivalis* PorLM, for which a lower-resolution map was obtained) was determined recently using cryo-EM (Figure 3C; Hennell James et al., 2021). The composition of the type IX secretion system and putative functions of its individual parts are described in a recent review (Gorasia et al., 2020). Like MotAB, GldLM consists of proteins expressed from a single operon (Braun et al., 2005). GldL and GldM form the outer and inner parts, respectively, corresponding to MotA and MotB. Like MotB, the periplasmic domains of GldM dimerize. However, GldLM shares no obvious sequence homology with MotAB. While MotB binds peptidoglycan, anchoring the complex, the periplasmic part of GldM contains four domains, which upon dimerization form an 18 nm long shaft that may function to transfer mechanical energy to a proposed GldNK ring that is required for gliding motility and Type 9 protein secretion (Leone et al., 2018). Transmembrane GldL subunits are arranged in an asymmetric pentagon, similarly to MotA in Gram-positive bacteria (Figures 3B,C). Potential causes and effects of GldLM asymmetry have been discussed (Hennell James et al., 2021).

## Phylogenetics

The evolution of proton-driven rotation appears to be functionally convergent between BFM stator units and rotary ATP synthases. These systems utilize the same principle of proton-driven rotation despite having separate evolutionary histories and seemingly unrelated amino acid and DNA sequences. However, within the 5:2 motors, divergence seems more likely, given the sequence and structural relationships between different systems. MotAB and ExbBD are known to be homologs (Marmon, 2013). MotAB and ExbBD and the other 5:2 motors TolQR and AglRQS, and GldLM not only serve different purposes; they also vary in how they are arranged within their larger systems. MotB, the inner part of the MotAB 5:2 motor, binds the peptidoglycan cell wall, anchoring the stator unit in place. By contrast, in current models of the function of GldLM, the inner GldM part rotates to pass mechanical energy to the rest of the gliding system. There is no direct evidence for anchoring of GldL, but without it rotation of the 5:2 motor could be wasted as the rotation of GldL in the membrane rather than transferred efficiently to the intended targets *via* GldM. It remains to be determined experimentally whether pentameric ExbB and GldL rotate in the membrane and, if not, which factors prevent them from doing so. Anchoring could be achieved either by binding an unknown protein partner, by alterations in local lipid composition, or even by lipid conjugation to the pentameric barrels. In the absence of structural information for TolQR and AglRQS, speculation as to their wider function is based on the assumption that they too are 5:2 rotary motors.

**Figure 5:**
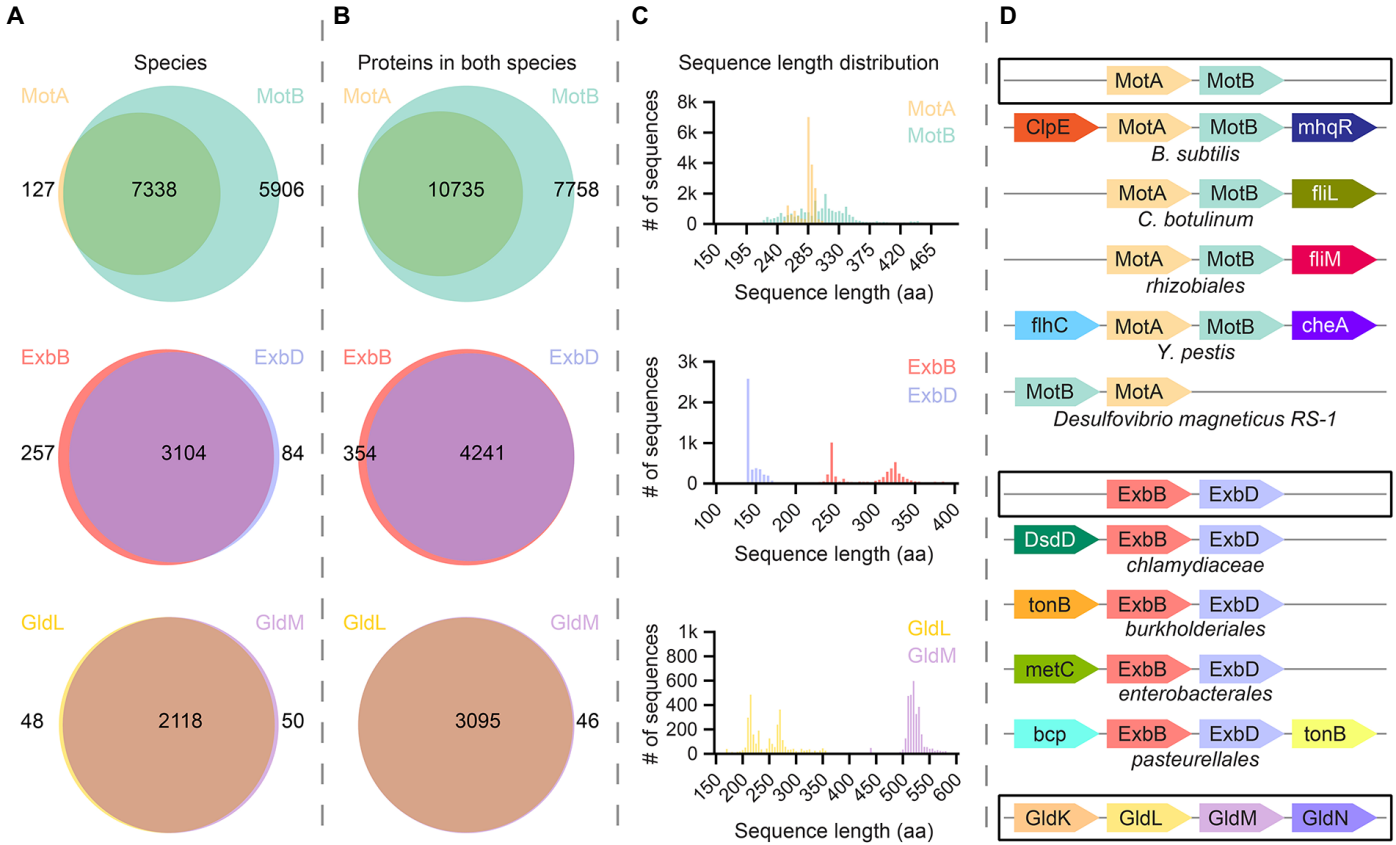
Bioinformatic analysis of bacterial proteins in the MotA/MotB family. **(A)** UniProt database were systematically screened for species that contain the MotA, MotB, ExbB, ExbD, GldL, or GldM proteins. In most cases, species contain annotations for both the transmembrane-barrel component and the rotational-axis component of a protein pair. **(B)** We analyzed the number of proteins in species that contain two elements of molecular motors [overlapping in **(A)**]. The number of annotated ExbB/D and GldL/M proteins is rather similar, whereas there are many more annotations for MotB than for MotA. **(C)** Sequence length distribution of all proteins described in **(B)**. Each histogram bin represents 5 amino acids. Proteins from the MotA family show a narrow two-peak distribution, whereas proteins from MotB family show more divergent lengths. ExbB and GldL proteins come in two length classes with normal distributions, while the size distribution of ExbD and GldM proteins is rather uniform. **(C)** The most prominent cases of synteny (co-localization) of genes encoding molecular machines. A typical scenario is highlighted by black rectangles. Functionally relevant or widespread genomic neighbors are shown. MotA-MotB synteny is broken only in the case of *Desulfovibrio magneticus RS-1*.

To explore the likely range and variation among 5:2 rotary motor complexes, we systematically queried UniProt database. First, we asked how many different bacterial species and strains possess individual MotA, MotB, ExbB, ExbD, GldL, and GldM annotated subunits. As expected, the majority of analyzed species contain InterPro family annotations for both complex-forming proteins (Figure 5A). In species that have 5:2 complexes with outer and inner proteins (overlapping circles in Figure 5A), some species contain multiple sets of outer proteins, which results in a greater number of proteins than species (Figure 5B). The number of species containing MotB is greater than those containing MotA (Figure 5A, top), and the number of MotBs in species containing both MotA and MotB is also larger (Figure 5B, top). This effect is absent, or even slightly reversed, for ExbBD and GldLM (Figures 5A,B, middle, bottom). This may simply be an artifact of poor protein family annotation (we examined five species with MotB but no MotA annotated, selected at random, and in all cases found a MotA gene candidate in the vicinity that might yield a protein product of high similarity to MotA). More interestingly, it may indicate various specialized MotA variants that assemble on-demand around a particular type of MotB protein that confines the stator to a particular molecular neighborhood. To our knowledge, there is no experimental evidence to corroborate or falsify this hypothesis.

To explore the variability of the proteins we identified, we analyzed the sequence-length distributions of proteins (Figure 5C). MotA proteins show two unequal, narrow peaks in the vicinity of 250 a.a. ExbB and GldL are very similar. The inner parts, MotB, ExbD, and GldM, each show a single peak, which is broad for MotB and narrow for the others. Unlike MotA, in which a relatively large portion of the protein is transmembrane, most of the polypeptide in the MotB family is periplasmic, and variation in the thickness of the periplasm between species may explain the broad sequence length distribution of MotB. ExbD is rather short compared to other inner proteins, and the distribution of sequence lengths of ExbD-like proteins shows a skewed single-peak distribution, which could be related to its interaction with TonB. GldM, by contrast, is much larger, which is consistent with its proposed function as a shaft spanning the periplasm.

Finally, we performed synteny (co-localization) analysis of genes encoding these 5:2 rotary molecular motors using GeCon T (Figure 5D; Martinez-Guerrero et al., 2008). The gene order appears to be well preserved in Bacteria, except for *Desulfovibrio magneticus* RS-1, in which MotB precedes MotA. Other interesting cases of the genomic context of stator unit genes are highlighted.

We tried to systematically check several model organisms for the existence of 5:2 rotary motor proteins using both annotations from UniProt database and protein BLAST to verify protein identity. Within our selected set of model species, MotA and MotB are present in all Gram-negatives and some Gram-positives. The ExbBD system is considered a property of Gram-negative bacteria, although there are several ExbB-family proteins annotated in *Staphylococcus aureus* and *C. sporogenes*. It remains to be tested experimentally whether, and if so how, some orthologs of the Ton system function in Gram-positive microorganisms with a thick cell wall and in the absence of the ExbD subunit. The GldLM system is present exclusively in Bacteroidetes.

## A model for the rotary mechanism

Any molecular motor is defined by coupling directed mechanical motion, on a length scale similar to that of the entire motor, to an energy source such as a chemical reaction. Coupling consists of a “mechanochemical cycle” – a cyclic set of transitions between different states that both consume (electro) chemical free energy and generate directed movement (Berry, 2000). The mechanism is defined by these states and by the constraints imposed by the structure of the motor upon transitions between them. In particular, for ion-driven rotary motors, binding or unbinding of ions must change the freedom of one part to rotate relative to the other, and the rotation angle must in turn change the accessibility of the ion-binding sites from each side of the membrane. It is the interplay of these constraints that defines the mechanochemical cycle.

**Figure 6:**
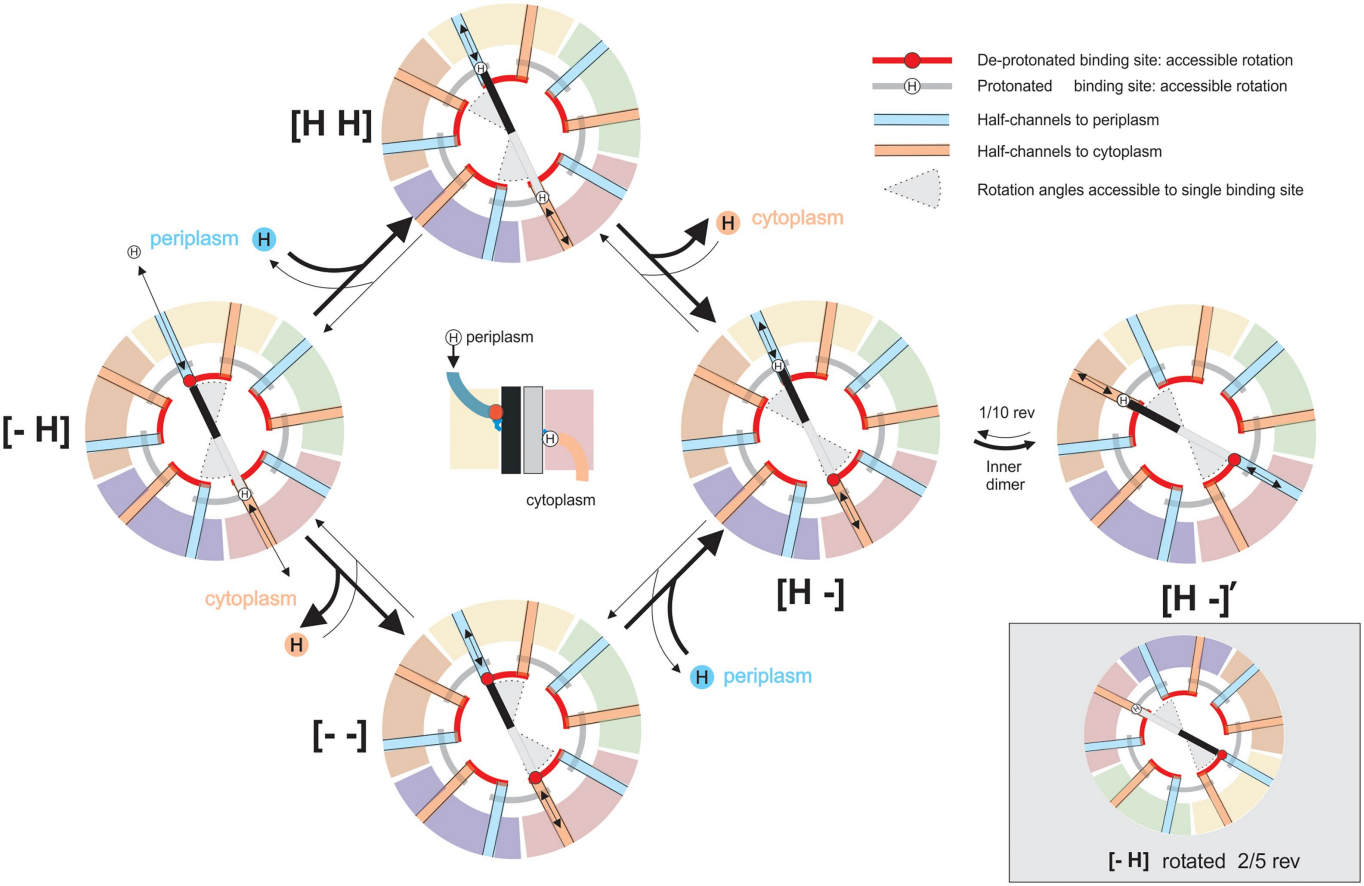
A model for the mechanistic basis for the function of 5:2 rotary motors. Schematic cross-sections of a 5:2 rotary motor in a membrane slice containing the proton-binding sites on the inner dimer. Different outer subunits are shown as arcs, distinguished by color, inner subunits are shown as black and gray bars. Deprotonated sites are negatively charged (red circles), protonated sites are neutral (“H”). “Half-channels” linking sites to the periplasm (blue) or cytoplasm (orange) are shown as colored radial lines. The location of half-channels relative to the boundaries between outer subunits is drawn from the MotAB structure (Santiveri et al., 2020), but it does not affect the mechanism and is speculative and illustrative only. Black arrows indicate transitions in the mechanochemical cycle, accessible from each state. Red and gray arcs indicate angles that a binding site can occupy when de-protonated or protonated, respectively. The central image shows a side view of state [− H], illustrating half-channels. In state [− H], the charged “black” binding site can bind a proton from the periplasm, and the “grey” site has a proton bound which can be released into the cytoplasm. Either of these transitions locks dimer rotation due to the constraints on individual binding sites (states [H H] or [− −]). After both transitions are completed the inner dimer is free to rotate counterclockwise (states [H -], [H -]’). States [− H] and [H -]’ are equivalent: rotation of the entire complex and exchange of nominally identical subunits maps one onto the other (bottom right). The cycle is reversible, but it is biased by the PMF, which drives protons from the periplasm to the cytoplasm.

The new structures of 5:2 rotary motors provide strong evidence for a rotary mechanism in two ways. Most obviously, the 5:2 symmetry mismatch between outer and inner parts creates a 10-fold periodicity in the rotation angle: states separated by 1/10th of a revolution are identical except for the exchange of individual, nominally identical, subunits. Thus, the mechanochemical cycle is fully defined by a sub-cycle that describes a 36° rotation. Figure 6 shows the simplest and most general such cycle for any 5:2 rotary motor, a variant of the classic “turnstile” model (Berry, 2000) first proposed for the entire BFM in the 1980s (Mitchell, 1984) and now believed to apply to the rotation of F_O_ of the ATP-synthase (Pogoryelov et al., 2009). The model is based on the following two critical constraints.

1. It is impossible for a proton-binding site (e.g., MotB-D22 in *C. jejuni* MotB) to access both periplasm and cytoplasm in the same mechanochemical state. Otherwise, protons could cross the membrane uncoupled from rotation – the motor would leak. Because 5 divided by 2 is not an integer, each proton-binding site contacts a different part of the inner cavity of the surrounding pentamer; allowing each to be accessible to different ionic “half-channels” in the pentamer. A half-channel has access to either the periplasm (e.g., Figure 2C) or cytoplasm. In the model presented in Figure 6, periplasmic and cytoplasmic half-channels are shown as thick blue and orange lines, respectively, and proton leakage is prevented by a rotational offset between the two sets of five channels. The offset angle is unknown, but for simplicity, Figure 6 shows the offset as 1/10th of a rev. The structures of the wild-type and protonation-mimicking D22N mutant MotAB (Figure 2C) suggest that motions of the side chain of D22 may also contribute to the separation of the cytoplasmic and periplasmic half-channels (Santiveri et al., 2020).

2. The central dimer (e.g., MotB_2_) is free to rotate within the pentamer, through ~1/10th of a revolution (between states [H -] and [H -]′ in Figure 6), only in a sub-set of protonation states. In Figure 6, a suitable set of such rotation constraints arises from simple constraints on individual binding sites: charged sites can access only angles indicated by red “rails,” whereas neutral protonated sites can access only angles indicated by gray rails. Santiveri et al.’s (2020) model based on details of the structure of MotAB imposes slightly different constraints, allowing rotation only when both MotB-D22-binding sites are protonated. A hydrophobic patch on MotA, which a charged site cannot pass, has been proposed as a possible structural explanation (Santiveri et al., 2020). The BFM is believed to work with a “power stroke” mechanism (Berry and Berg, 1999; Lo et al., 2013), which would be the case if the rotation transition releases free energy, for example by coupling proton motion across some part of the membrane voltage to rotation (Santiveri et al., 2020). In general, however, this rotation need not be directly coupled to proton motion in all 5:2 rotary motors.

Figure 6 illustrates the mechanochemical cycle that couples the net transport of one proton across the membrane to a rotation of 36°. Individual MotA subunits of the outer pentamer are identified by different colors, and the inner dimer molecules are shown in shades of gray and black. The transitions out of each state that are allowed are indicated by black arrows. The range of accessible rotation angles (gray pie-wedges) rotates by 36° when the protonated binding sites are swapped, between [H -] and [− H], in a direction that depends upon the direction of proton flow that achieved the swap. The heavy arrows in Figure 6 show the preferred directions with a typical PMF, which drives proton influx. The turnstile cycle can be understood by following the proton that binds to the “black” site from the periplasm in state [− H], rotates around the gray rail from a periplasmic half-channel to a cytoplasmic half-channel, i.e., from [H -] to [H -]′, and leaves to the cytoplasm. Protons on the gray site follow an equivalent path but are half a cycle out of phase. It is the net left-handed helicity of these paths that sets the direction of coupling between rotation and proton flux, in this instance coupling proton influx to CW rotation of the pentamer relative to the dimer, as viewed from outside the cell. Reversing the direction of proton flow to outward in non-switching chemotaxis mutants, either by setting up an interior-positive K^+^-diffusion potential in *Streptococcus* strain V4051 (Berg et al., 1982), or by reversing the polarity of voltage-clamped giant *E. coli* cells (Fung and Berg, 1995), appears to reverse the direction of motor rotation.

There is currently no evidence as to whether the ion-binding sites (and channels) in GldLM are on the inner or outer components. The turnstile model can accommodate either possibility; it requires only that the half channels and binding sites are on different components and that they describe “helical” ion transit paths that include rotation between inner and outer components. It is not known in any 5:2 motor whether protonation swapping proceeds *via* doubly protonated [H H] or doubly charged [− −] states, or both, in part because current resolution of the obtained cryo-EM reconstructions does not allow visualization of protons.

The mechanochemical cycle of Figure 6 does not include a role for the experimentally observed broken C_2_ symmetry of the MotB dimer within the entire 5:2 complex, nor for conformational changes within the dimer or pentamer (Kojima and Blair, 2001). However, the general scheme (Figure 6) still works in essentially the same way if the central dimer is allowed to have multiple conformations and to sit asymmetrically within the pentamer, for example, following the Santiveri et al. model (Santiveri et al., 2020; Supplementary Figure S1). The details of motor constraints should be revealed as new structures capture new states in the cycle and biophysical experiments constrain models according to their predicted patterns of flagellar rotation.

Various cations can replace H^+^ as the coupling ion in the BFMs of different species. For example, some Na^+^-driven stator units also work with Li^+^ or Cs^+^. With its high atomic number, Cs^+^ is a good candidate for potential structural determination of the ionization states of the motor. Whatever details may be discovered in various 5:2 rotary motor mechanisms, 10-fold rotational symmetry is almost certain to be a universal feature.

The rotary model is very a natural inference from the structure of 5:2 motors. The present state of knowledge is comparable to that of F_1_ ATPase after the first atomic structure of F_1_ (Abrahams et al., 1994) showed 3:1 symmetry, with a 3-fold α_3_β_3_ outer symmetry broken by the asymmetric inner γ-subunit, strongly suggestive of a rotary mechanism. Direct proof of the rotary mechanism by single-molecule biophysical experiments soon followed (Noji et al., 1997).

What does the rotary model of 5:2 motors predict for such experiments? First – rotation itself, confirming that defining feature of the mechanism. Second – the mechanochemical cycles described above couple the transit of 10 ions to each rotation of the motor. This sets an upper limit of *10ePMF/2π* to the motor torque, *via* the principle of Conservation of Energy, where *e* is the unit charge and *ePMF* is the free energy available per ion transit. Third – rotation is predicted to occur in 36° steps corresponding to transitions between symmetrically equivalent states ([− H] and [H -]′ in Figure 6), one per ion transit. The symmetry between these equivalent motor states is broken if the 5:2 rotary motor interacts asymmetrically with an external component. For example, if the flagellar C-ring is to the left in Figure 6, then the two MotB chains are no longer equivalent. In the current model of the BFM, MotA_5_ interacts with the C-ring at its proximal side during CCW rotation and at its distal side during CW rotation (although recent studies show that a smaller-diameter C-ring induces CW rotation (Sakai et al., 2019), questioning this model). If these interactions are very similar, the effects of C-ring-induced asymmetry may be minimal. Nonetheless, in the interactions with the C-ring, there are only 5 equivalent states per rotation, which may occur in five 72° steps or by “limping” with five pairs of 36° steps with different dwell times. If they could be measured, these patterns of rotation and their dependence upon external factors such as ion concentrations and ion motive force would allow confirmation and a full understanding of the rotary mechanism.

## Functions as components of larger systems

**Figure 7:**
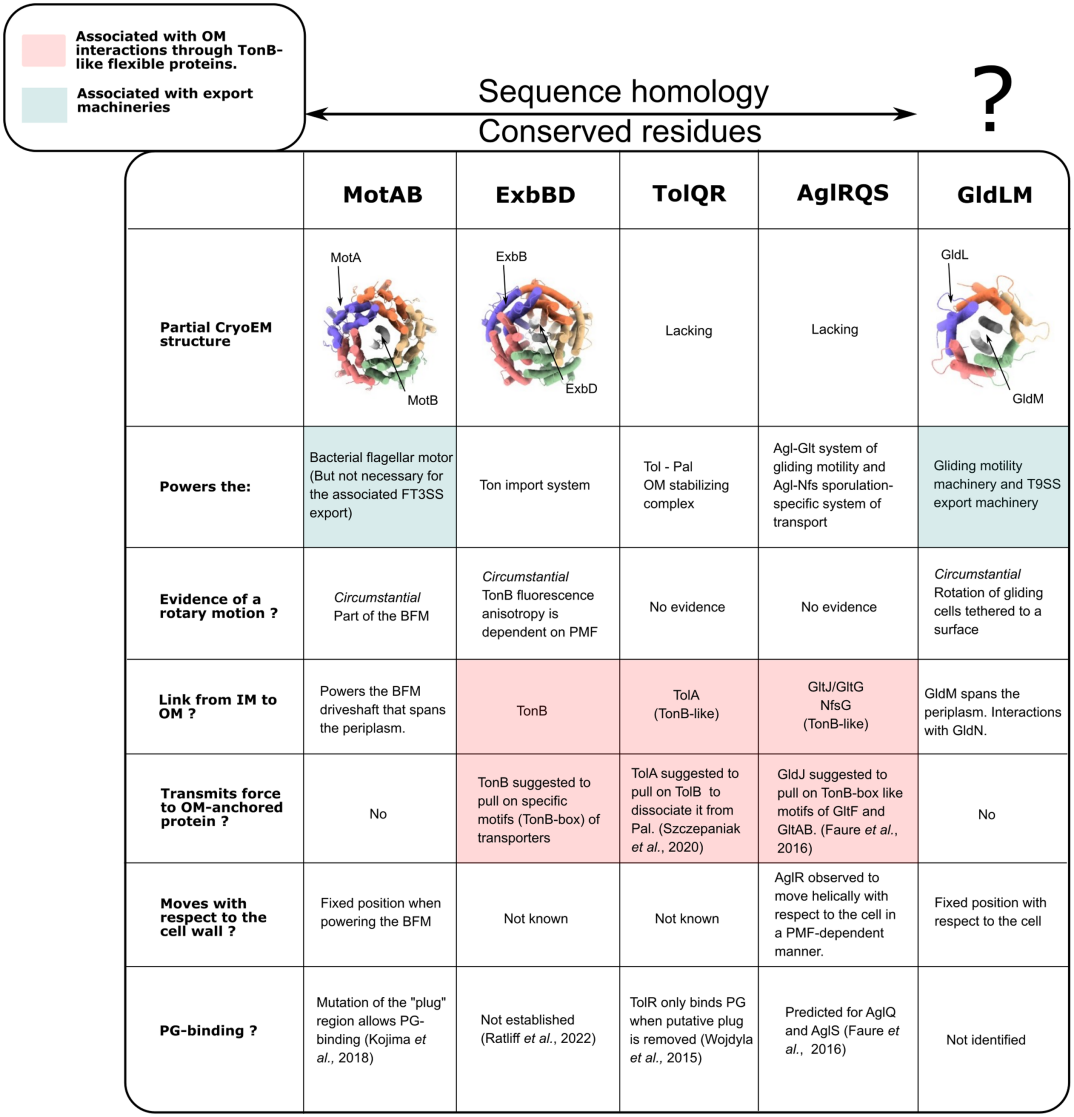
Functional comparisons of the PMF-dependent bacterial complexes with known 5:2 structures (MotAB, ExbBD, and GldLM) and their sequence homologs. PDB ID for CryoEM structures, from left to right: 6YKP, 7AJQ, 6YS8. GldLM is reported as lacking sequence homology with other 5:2 motors. However a pairwise sequence alignment using EMBOSS Needle and BLOSUM62 showed identity and similarity of ~15 and 31%, respectively, for MotA/GldL, 18 & 30% for MotB/GldM, indicating the possibility that they might be homologs.

To date, high-resolution structures of 5:2 rotary motors have been obtained in three different bacterial systems, and homologous proteins and lower-resolution structures are known in a further two systems (Figure 7). It is reasonable to assume that all share the same essential structural and mechanistic features. Here we discuss what is known, or can be speculated, about how the rotation of the 5:2 motor is coupled to its function in each system.

### MotAB, bacterial flagellar motor

The BFM (Figure 1) is the best understood of these systems, and the only one in which rotation of any component (here, the BFM rotor) has been conclusively demonstrated. The recent MotAB structures, along with cryo-electron tomography (ET) of entire BFMs *in situ* (Zhao et al., 2013; Carroll et al., 2020), have overturned previous ideas about how the BFM works. The new picture is that energy transduction from the ion motive force to torque occurs entirely within the MotAB stator units, which act as small wheels to rotate the much larger C-ring. The long-established rotation of the BFM is the main supporting evidence for the rotary model of 5:2 complexes. Further support is provided by recent structures of the entire flagellar motor of *Borrelia burgdorferi*, obtained *in situ* by cryo-ET (Chang et al., 2020). These images show that the C-ring contacts opposite sides of the stator units in flagellar motors that are believed to be rotating in opposite directions (Figure 1B). This would allow unidirectional rotation of the stator units to power rotation of the flagellar motor in either direction, explaining switching in the direction of flagellar rotation, which is at the heart of bacterial chemotaxis. Considerable uncertainty remains as to the details of this model of flagellar switching. In *B. burgdorferi* (Chang et al., 2020), CW motor rotation is associated with increased radius at the top of the C-ring, implying CW rotation of MotA_5_ (Figure 1B). But in *Vibrio alginolyticus* (Carroll et al., 2020), the change in C-ring radius associated with directional switching is too small to span MotA_5_, as required by the *B. burgdorferi* model. Furthermore, a FliF-G fusion-deletion mutant of *Salmonella enterica*, with a C-ring of reduced diameter and no apparent space for interactions between FliG and the distal part of MotA (Sakai et al., 2019), rotates CW. Therefore, it remains possible that the details of the flagellar switch, and possibly even the direction of rotation of MotAB, may vary between species.

The MotAB structures show the MotB “plug” helices (Hosking et al., 2006) bent down, possibly closing the periplasmic ion channel and also blocking rotation of the MotB dimer within the MotA pentamer (Kojima et al., 2009, 2018; Li et al., 2011; Terahara et al., 2017; Figure 2B). Studies of single motors show that MotAB stator units in *E. coli* are mechanosensitive – the number of active units is greater when the BFM is working against a higher load (Lele et al., 2013). This has been modeled as a catch bond, which has a decreased off-rate for binding when a load is applied (Nord et al., 2017a). Estimates of the force dependence of the off-rate are consistent with a transition state for activation of tight binding that is shifted by 4 nm from the loosely-bound state (Wadhwa et al., 2021). By comparison, if the C-terminus of the structurally resolved plug helix was pulled to its maximum distance from the membrane without unfolding, it would move about 3 nm from its closed position.

Taken together, these observations suggest a molecular model for mechanosensing. Force generated at the MotA-FliG interface when the motor works under load is transferred to the cell-wall anchor along the length of MotB. This pulls on the C-terminus of the plug helix, opening the ion channel and activating the stator unit and the catch-bond. This model also explains the recent observation that the first stator unit takes longer to bind than subsequent units (Ito et al., 2021). When the first (still plugged) unit binds to both the rotor and the cell wall, there is no motor torque to pull on the plug to activate the unit and start motor rotation, so activation is relatively slow. Subsequent units bind a motor that is already rotating, which pulls the plug as soon as it binds the cell wall, activating these units more rapidly.

Nothing is yet known about the details of the rolling interface between the small and big wheels in the flagellar motor. We can, however, speculate that a likely mechanism is a form of cog-wheel, in which each of 5 MotA “teeth” engages a matching structure in the FliG component of the C-ring. This is supported by the fact that the ratio of FliG units in the C-ring and of MotA units in the stator element (34/5 = 6.80) in *S. enterica* is close to the ratio of the radius of the C-ring and the radius of the MotA pentamer ring (21–25 nm/3.5 nm = 6–7) (Thomas et al., 2006; Chen et al., 2011; Santiveri et al., 2020). Thus, the tangential distances between adjacent FliG and MotA monomers are very similar. This would allow FliG and MotA monomers to match up during rotation. Even if the interface is softer than a cog-wheel implies, tight coupling between rotations of the two wheels is a reasonable expectation, with a gear ratio set approximately by their relative sizes.

Because the C-rings of *E. coli* and *S. enterica* have a 34-fold symmetry (Kawamoto et al., 2021), the cog-wheel model predicts 34/5 revolutions of each MotAB stator unit per revolution of the C-ring. Ten ions per revolution of the MotA pentamer then correspond to 10 × 34/5 = 68 ions per MotAB stator unit per revolution of the flagellar motor. (By the same reasoning, in *B. burgdorferi* in which the larger C-ring has a 46-fold symmetry, there would be 92 ions per revolution per stator unit.) Biophysical measurements of torque and speed in single flagellar motors in *E. coli* find a maximum torque of 0.9 pN nm/mV/stator unit, from which energy conservation sets a lower limit of 39 ions per revolution per unit (Nord et al., 2017b). This is less than the above prediction of 68 ions per rev, as required by energy conservation, but the difference between the two numbers begs an explanation – if the experimental estimates are all accurate, the system is wasting energy somewhere. It is also possible that published torque estimates are too low because they were obtained using viscous drag coefficients of polystyrene beads without considering the possibility of contact friction with the cell surface. Alternatively, if there are systematic errors in the opposite direction, then the lower limit might be 34 rather than 39 ions per revolution, corresponding to 5 rather than 10 ions per revolution of MotAB. This would require an entirely new model for the mechanism by which 5:2 rotary motors operate.

The model of 5:2 rotary stator units also makes predictions about the stepping patterns in flagellar rotation. The 26-fold stepping previously reported in the BFM (Sowa et al., 2005) is now understood to be due to the passive bearing at the LP-ring/rod junction (Johnson et al., 2021; Yamaguchi et al., 2021) and not as a signature of the torque generating mechanism. Because the C-ring further breaks the symmetry of the 5:2 MotAB rotary motor, either 10- or 5-fold stepping rotation of MotAB might be observable, corresponding to 68- or 34-fold stepping of the flagellar motor. However, if the coupling is loose – either between ion transit and rotation of MotAB, or in the interface between MotAB and FliG, then very different or even no stepping patterns might be seen. Kinetics of 34-fold steps might contain a signature of underlying 68-fold symmetry, with two proton transits powering each 34-fold step; by direct analogy with unresolved transitions in the F_1_ ATP-hydrolyis sub-step [first described as 30° (Yasuda et al., 2001), then revised to 40° (Ueno et al., 2005)]. These two sub-steps were later assigned to separate events in the ATP hydrolysis cycle. The BFM is reviewed elsewhere in this collection (Manson, 2022).

### How does current biophysical knowledge of other 5:2 complexes accommodate a rotary model?

The rotary model of MotAB naturally matches its function, powering the rotary BFM. How the known functions of other 5:2 rotary motors (Figure 7) might be achieved by rotation is much less clear. Published biochemical and biophysical characterizations allow the formulation of some hypotheses, which we discuss here.

### ExbBD, Ton import system

Fluorescence anisotropy measurements (Jordan et al., 2013) suggest that the ExbBD-bound protein TonB undergoes reorientation *in vivo* during the fluorescence lifetime of the labeling GFP, dependent upon PMF and ExbBD. This is weak evidence for rotation of ExbBD, given that *any* movement of the protein is likely to cause its reorientation. However, it confirms the motion of TonB in the IM coupled through ExbBD to the PMF. As reviewed in the introductory sections, ExbBD powers the unplugging of OM-embedded TonB-dependent transporters (TBDT) *via* the interaction of TonB with the “TonB-box” domain of TBDT. The TBDT thus acts as a gate that can be opened by the PMF, whose energy is harvested by ExbBD, through the action of TonB. TonB is a long protein that spans the periplasm. It has a short N-terminal domain in the cytoplasm, followed by a single transmembrane (TM) helix that interacts with ExbB (Higgs et al., 2002) and a periplasmic domain composed of a flexible linker and a folded C-terminal domain. Single-molecule studies of the unfolding of the plug domain of the TBDT by itself (Thoma et al., 2012) or mediated by TonB (Hickman et al., 2017) showed that forces of at least 50–60 pN were necessary to unfold the TonB boxes. This energy must be transmitted by TonB. TonB is energized by the IM-embedded ExbBD complex and must span the periplasm to interact with the OM-embedded TBDT. This raises two questions: how does TonB find the TBDT and how does it exert force on it?

Regarding the first question, one can formulate several hypotheses. TonB and ExbBD must be recruited to the vicinity of the TBTD and bind to the cell wall there. This process is similar to the dynamic recruitment of MotAB to the BFM (Leake et al., 2006). It is important to note, however, that the TonB-ExbBD complex cannot diffuse in the IM while TonB crosses the PG, as the PG is a solid structure with ~2 nm pores (Demchick and Koch, 1996). It is possible that ExbBD and TonB find the TBDT separately: either could take the lead and recruit the other. Alternatively, TonB might be able to retract to the IM side of the periplasm while bound to ExbBD, allowing IM diffusion of the whole complex. This is supported by NMR data showing that the central portion of TonB consists of two rods spanning 10 nm and joined by a short flexible sequence (Evans et al., 1986; Brewer et al., 1990). This short sequence could allow the extended TonB to fold back on itself in the IM-periplasmic space. The ExbBD-TonB complex could then diffuse, with TonB periodically extending to cross the cell wall to sample the OM until it finds a TBDT. It is interesting to note that ExbD has been shown to form a heterodimer with TonB *in vivo* (Ollis and Postle, 2011) through the same domain of ExbD (structurally similar to the PG-binding motif of LysM (Klebba, 2016), which can homo-dimerize). This suggests that the PG-binding of ExbBD could be induced by TonB and its interaction with the TBDT.

**Figure 8:**
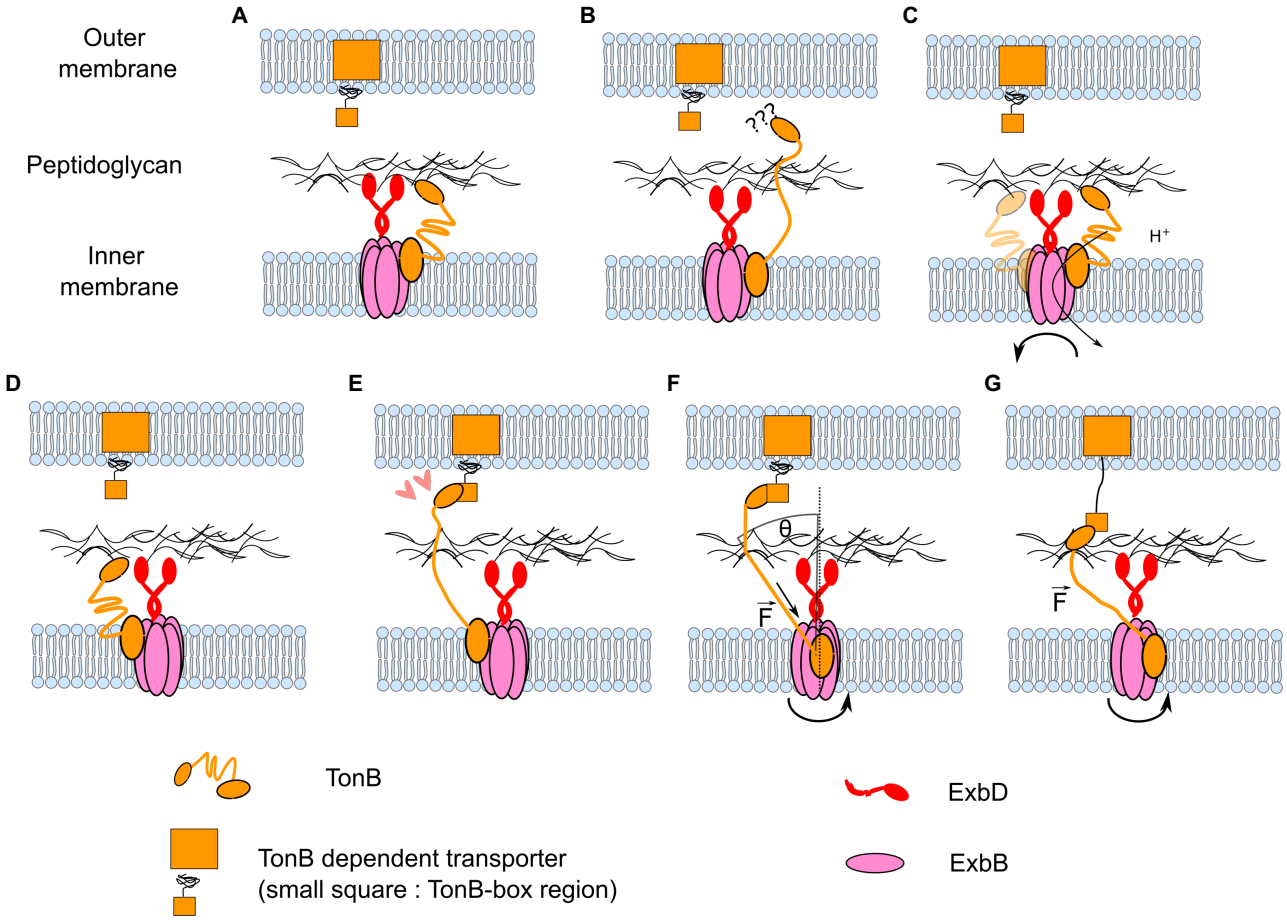
Model for how rotary motion of ExbBD could accomplish the functions of seeking TonB-dependent transporters and pulling on them. **(A–C)** Coupled with the flexibility of TonB, the rotation of ExbBD would allow the repositioning of TonB when it is retracted into the IM periplasmic space. **(D–E)** When it is properly positioned, TonB can bind to the TonB-dependent transporter. **(F)** Rotation of ExbBD pulls on the N-terminus of TonB. The maximum force applied to TonB depends on the angle θ and the number of ions per turn *N_r_*. **(G)** A sufficiently high force partially unfolds the TBDT and allows the import of extracellular molecules.

It then remains to explain how ExbBD-TonB can exert force on the TBDT. Klebba proposed that rotation of TonB might be involved in harnessing the PMF (Klebba, 2016). However, at that time there was no hint that ExbD might rotate relative to ExbB. Here, we propose a model in which rotation of ExbBD exerts a force on TBDT *via* displacement of the N-terminus of TonB (Figure 8). The free energy per proton depends on the PMF (∆*G* = *ev*), which in *E. coli* is about 25 pN.nm. The external radius of ExbBD is about 4 nm. In the rotary model ExbBD uses 10 ions per revolution. This allows a rough estimation of the tangential force (F) that ExbBD can apply at its external limit: $F=\frac{N_{p}\Delta G}{2\pi R}=10\;pN.$ Because TonB is anchored to the peptidoglycan and is not contained in the plane of the inner membrane, the maximum force applied on TonB is $F_{\max}\sim \frac{N_{p}\Delta G}{2\pi R\;\sin\left(\theta \right)},$ where θ is the angle between the direction of TonB and the normal to the plane of the inner membrane. One may note that in this model, the force will only be transmitted to the TonB-box: 1. if the space between the IM and OM is rigidly maintained; 2. if TonB is tensioned enough. Regarding the second point, TonB can always be tensioned by progressively wrapping around ExbBD until it reaches the right extension.

A general feature of this mechanical scheme is a high gear ratio: relatively small torques can exert very large forces if θ is small. Combined with the possibility of unlimited rotation, this allows the energy from many protons to be harnessed to pull hard and far on the linear element. In this case, a pulling force of 50 pN can be achieved as long as θ < ~10°, suggesting that such a rotate-and-pull mechanism is plausible. The Ton system is reviewed elsewhere in this collection (Ratliff et al., 2022).

### TolQR, Tol-Pal system

Although the structures of individual components are known, the structure of the overall TolQR complex, which shares sequence homology with other 5:2 rotary motors, is still missing (Abergel et al., 2001; Zhang et al., 2009; Szczepaniak et al., 2020a). TolR is predicted to contain a single TM helix with a conserved aspartate mid-way across the membrane. A structural model for the assembly of TolQR in a 5:2 stoichiometry, based on the structure of ExbBD, has been recently proposed (Szczepaniak et al., 2020b). TolQR is a MotAB-like complex that is essential for the function of the PMF-dependent Tol-Pal system (Germon et al., 2001; Witty et al., 2002; Goemaere et al., 2007). One of its many functions is to stabilize, through PG-binding of the OM lipoprotein Pal, the outer membrane at the septal site during bacterial cell division.

The stability of the outer membrane decreases and its porosity increases in Δ*pal* mutants. Much like TonB associates with ExbBD in the Ton system, the TolA protein can span the periplasmic space and interact at its N-terminus with the TolQR IM motor complex (Koebnik, 1993) and interact at its C-terminus with the outer membrane protein TolB. TolB inhibits the binding of Pal to the PG (Bonsor et al., 2009). Recent data (Petiti et al., 2019) show that, during cell division, TolQ proteins localize close to the septal site even in ΔTolA, ΔTolR, Δ*pal*, and ΔTolB mutants, and TolA proteins localize similarly even in ΔTolQR, ΔTolB, and Δ*pal* mutants. Pal also localizes at the division site, but only in cells expressing TolA and functional TolQR.

From these data, Petiti et al. propose that TolQR is first recruited to the septal site and then energizes and extends TolA through the periplasmic space to the OM. TolA then recruits TolB and Pal. The extension/retraction of TolA would be facilitated by the hairpin structure of its periplasmic domain. However, Szczepaniak et al. (2020a) showed that during cell division, when TolQR is recruited to the septal site, diffusion of Pal away from the division site increases compared to non-dividing cells. From this observation, combined with the knowledge that TolB inhibits PG-binding by Pal, they propose another model in which the energization of TolQR causes TolA to extend through the PG layer, bind to TolB, and pull on it to disassociate it from Pal (Szczepaniak et al., 2020b). Pal would then bind to the PG, explaining why it concentrates where TolQR is recruited. This model, with TolA pulling on TolB powered by PMF energization of TolQR, is similar to the opening of the TBTD by TonB, and thus also fits the seek-then-pull model described in Figure 8, including a rotary mechanism for TolQR. The Tol-Pal system is reviewed elsewhere in this collection (Webby et al., 2022).

### AglRQ/S, adventurous (A) gliding system

The essential members AglRQ/S of the PMF-dependent adventurous (A) gliding machinery of *Myxococcus xanthus* also present sequence similarities with the stator units of the BFM: AglR is homologous to MotA, AglQ and S are homologous to MotB and are predicted to contain a single TM helix with a conserved aspartate mid-way across the membrane, and both AglRQ and AglRS can theoretically form MotAB-like proton channels (Sun et al., 2011). AglR (Nan et al., 2013; Fu et al., 2018) and AglZ, a protein that co-localizes with AglQ, (Faure et al., 2016) were shown to follow helical trajectories. Furthermore, when the PMF is blocked, AglR stops moving (Nan et al., 2013). AglQ was shown to accumulate at focal adhesion points (Faure et al., 2016) with AglZ, where it stops moving relative to the extracellular matrix (ECM) and co-localize with the outer membrane components of the A-gliding machinery, GtlCD. The formation of such static clusters, which keep moving relative to a fixed marker on the cell wall, but are fixed relative to the ECM, is associated with the cell starting to move helically and gliding. From those data, a model was proposed in which AglRQ/S would power gliding by anchoring to the cell wall *via* the TolR-like PG-binding domains of AglQ/S (Wojdyla et al., 2015) and exerting a force between the cell wall and OM-bound proteins.

In this model, AglRQ/S would be able to anchor to the cell wall to exert a force on OM-bound proteins but also move relative to the cell. One can formulate two hypotheses to explain this observation. AglRQ/S could be anchored to a fixed patch of the PG, requiring dynamic re-polymerization of the PG to move relative to the cell. This seems to be excluded by data that show that antibiotics that block PG-polymerization do not affect the helical movement of MreB, the latter being dependent on AglQ/S and on the PMF (Fu et al., 2018). Alternatively, AglRQ/S could form complexes that move processively along the cell wall, in the same way that kinesin walks on microtubules but powered by PMF.

**Figure 9:**
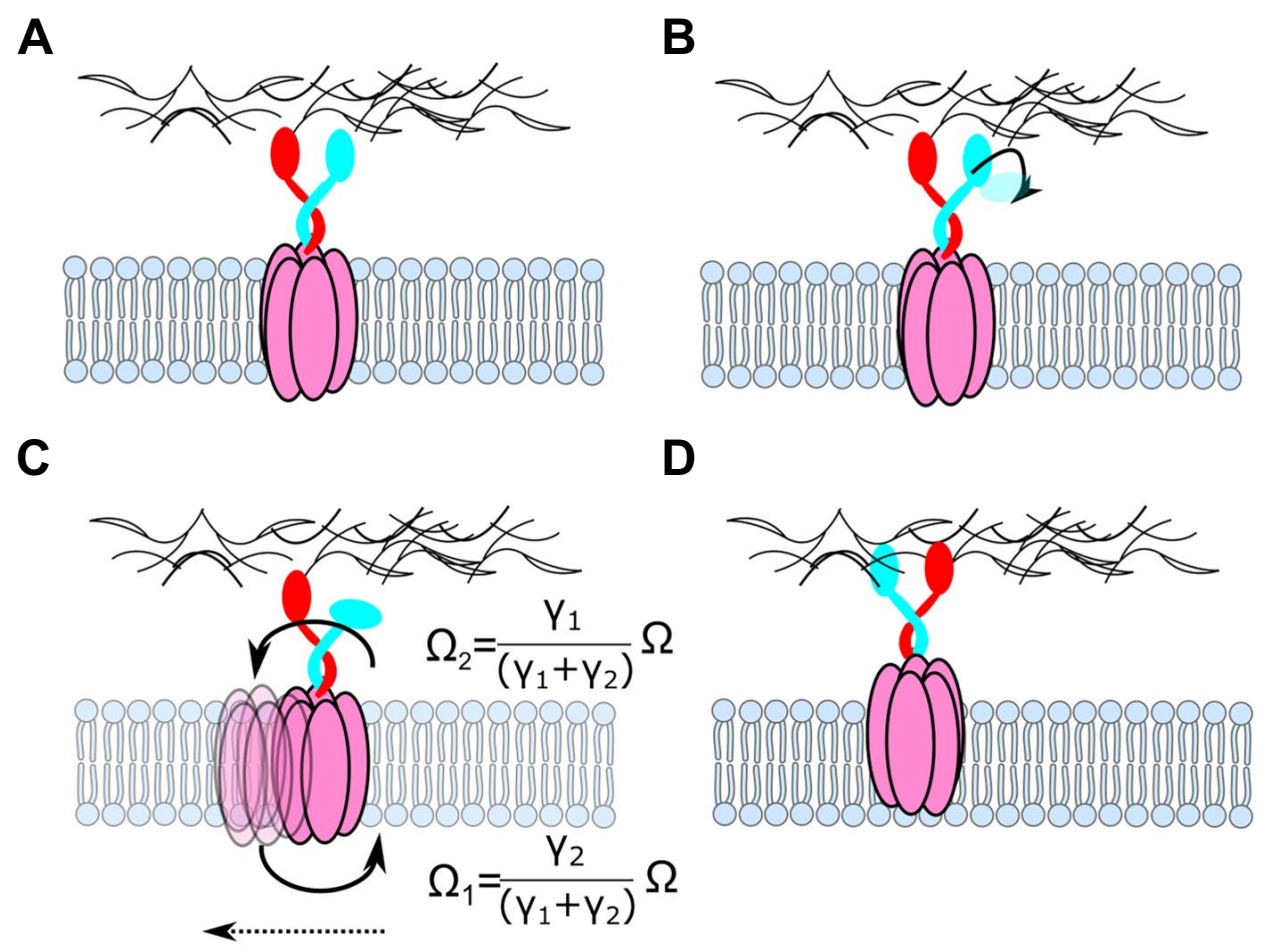
How rotation of a 5:2 AglRQ/S complex could accommodate the observed movement of AglR relative to the cell wall. **(A)** The two inner units are attached to the peptidoglycan. **(B)** One unit detaches from the cell wall. **(C)** Because both the dimer and the pentamer are free to rotate, PMF-driven rotation of one complex relative to the other would cause them to rotate in opposite directions, with angular velocities (Ω) depending on the ratio of the drag coefficients (γ). The detached domain binds again to the peptidoglycan, but it binds at a different location.

How could these results accommodate the hypothesis that AglRQ/S also forms 5:2 rotary motors? It is noteworthy that kinesins, although through completely different mechanisms, move by transforming a rotation between two anchoring points into translation (Asbury et al., 2003; Yildiz et al., 2004; Ramaiya et al., 2017). Figure 9 illustrates a model for AglRQ/S that works in the same way. In this model, however, it would be hard to account for the AglZ-dependent regulation of the direction of movement by the Frz pathway (Zhang et al., 2012; Nan et al., 2013). Other components would be necessary to bridge the gap between 36° random rotation and directed translation along helical paths. On the other hand, the similarity of GltG/GltJ to TonB (flexible linkers, TonBC-like motifs) and of GltF with the TonB-Box (Faure et al., 2016) is compatible with the same type of pulling model as for the Ton and Tol-Pal complexes. Rather than ExbBD opening the TBDT by pulling through the cell wall *via* TonB (Figure 8), AglRQ/S would pull on the OM adhesion point through the cell wall *via* GltGJ and move the cell wall towards the adhesion point to induce gliding. The *Myxococcus* gliding system is reviewed elsewhere in this collection (Chen and Nan, 2022).

ExbBD, TolQR, and AglRQ/S are thus possibly structurally and functionally similar 5:2 rotary motors that use PMF-driven rotation to pull on an OM-embedded component by using a flexible linker that spans the periplasmic space. All of these systems would benefit from the high gear ratio that results from pulling *via* rotation, as described above for ExbBD (Figure 8). This hypothesis suggests further experiments to understand the dynamics of their PG-binding. In particular, further measurements of the PG-binding efficiency of the various complexes could shed light on the difference between static complexes (e.g., MotAB) and those that can move along the cell wall. Single-molecule trajectories of MotAB, ExbBD, and TolQR are lacking and would be interesting to compare with AglRQ/S. Determining the stoichiometry of the AglRQ/S complex *in vivo* will also be a critical step in understanding their function: AglQ and S are both homologous to MotB, but it is not yet known whether they can form heterodimers or homodimers in their complex with AglR.

### GldLM, bacteroidetes gliding system

In the case of the 5:2 GldLM complex, although there is no conclusive evidence of sequence homology with the other complexes, some biophysical results point towards a rotary mechanism. GldLM powers the gliding of *Flavobacterium johnsoniae* by inducing helical movement of the adhesin SprB relative to the cell (Shrivastava et al., 2016). Similar helical movements were seen in the 1980s in another gliding bacterium (Lapidus and Berg, 1982). However, cells sheared and attached to a slide coated with anti-SprB antibodies rotate (Shrivastava et al., 2015). Unlike AglRQ/S, GldLM is thought to be fixed relative to the cell wall while SprB moves (Shrivastava and Berg, 2020). This idea is consistent with the observation that dimeric GldM is longer than its equivalents in other 5:2 rotary motors (e.g., see Figure 5C), is less flexible, and may span the whole periplasmic space and cross the cell wall. These observations led to a rack-and-pinion model (Shrivastava and Berg, 2020), in which SprB is fixed to a helical track spanning the entire length of the cell that is set in motion along its length by rotating GldLM complexes that are anchored and distributed at various points over the cell surface. GldJ is a candidate for forming this track (Shrivastava and Berg, 2020), as it has been shown to decorate helical macrostructures (Braun and McBride, 2005). GldJ also was shown to interact with GldK (Johnston et al., 2018), which itself has been shown to form contacts with GldM (Vincent et al., 2022) and thus could serve to couple PMF-induced rotation of GldM to the movement of a GldJ track. GldM also forms contact with GldN, and GldK/N are suspected to form rings in the periplasmic space below the outer membrane (the formation of the ring has been shown for the homologous proteins PorK and PorN (Gorasia et al., 2016)). The fact that the periplasmic structure of GldM displays a strong bend led to the proposal that the tip of GldM describes a circle that could induce GldKN rotation (Hennell James et al., 2021). However, the measured diameter of the PorKN ring (50 nm) does not match the diameter of this circle (20 nm), suggesting that GldK/N would form smaller rings than PorK/N.

Overall, although a rotary mechanism for GldLM was proposed before the publication of the 5:2 structure, there is no direct evidence for rotation, and the proposed mechanism remains hypothetical. In particular, many proteins of the *spr* and *gld* operons, like GldA,B,D,F (Hunnicutt et al., 2002), GldI (McBride and Braun, 2004), and SprC,D (Rhodes et al., 2011) are necessary for the gliding-related spreading of colonies, but their role in the gliding machinery is not known. It is difficult to distinguish between proteins that are directly involved in the gliding mechanism and those that are involved in the associated T9SS export machinery. Indeed, in *F. johnsoniae*, gliding and the T9SS are intertwined because the adhesins necessary for gliding are themselves exported through the T9SS. Any defect in the export machinery would therefore cause a defect in gliding.

Gliding and export might be disentangled by adding the proteins necessary for gliding exogenously, but they would still need to cross the outer membrane, since many of them are located between the PG and the OM. In the case of the bacterial flagellar motor, rotation was observed by attaching beads to the hook or filament stubs (Ryu et al., 2000) that protrude outside the cell. However, in the case of the gliding machinery, the candidates for rotary motion, GldM, K, and N, lie inside the outer membrane, making them impossible to label with microparticles.

Another question is whether any protein complex of the gliding machinery might play the role of a drive shaft to transmit rotation of GldM through the PG, like the rod in the BFM. However, recent low-resolution cryo-ET images of a related Type-9 secretion system (Song et al., 2022) show no sign of such a shaft. If GldM rotates, the GldL pentamer must be anchored to the cell. This is another difference from the proposed mechanisms for MotAB, ExbBD, TolQR, and AglRQ/S, where the dimer is anchored to the cell wall and the pentamer rotates. The structure of GldLM does not leave space for GldL to anchor to the cell wall (Hennell James et al., 2021). Instead, the proposal is made that GldL is anchored to an as yet unidentified cytoskeletal protein through its long cytoplasmic tail.

All these questions will need to be addressed through advanced biochemical and biophysical measurements. In particular, *in vitro* reconstitution of single 5:2 complexes into artificial membranes and subsequent labeling with microspheres should confirm the rotary hypothesis and help understand the dynamics of these motors. *In vivo* orientation measurement through single-molecule polarization microscopy (Shroder et al., 2016) of fluorescently labeled proteins is another possible approach to circumvent the difficulty of labeling periplasmic proteins with microparticles. The Bacteroidetes gliding system is reviewed elsewhere in this collection (Trivedi et al., 2022).

## General considerations of rotary motor symmetry

Is there any advantage to the 5:2 symmetry of the motors described in this review over other possible symmetries? 5 and 2 have no common divisor >1. This property was discussed for the case of the passive bearing in bacteriophage rotary injection systems (Hendrix, 1978). It prevents several proteins in the inner part of the phage from being in the same position relative to those of the outer part at the same time. This flattens the energy landscape of interaction, since neither the most energetically favorable nor unfavorable positions can be occupied simultaneously by many pairs of proteins, avoiding very stable “traps” and high energy barriers, respectively. The lowest possible motor symmetries with no common divisors are 2:1, then 3:1 and 3:2, then 5:1 and 5:2. The 5:2 rotary motor symmetry may be preferred to lower symmetries due to packing difficulties or structural constraints. However, another important factor is the relationship between symmetry and the number of ions that the rotary machine uses per revolution. In a motor with *a:b* symmetry, each of the *a* outer proteins will pass each of the *b* inner proteins exactly once per rev. If each ion transit corresponds to one instance of a pair of inner and outer protein subunits passing any given relative angular position, as in all current models of ion-driven rotary motors, an *a:b* motor will use *a x b* ions per rev.

Following this reasoning, both 5:2 rotary motors and the 1:10 F_O_ motor of *E. coli* F_O_F_1_-ATPase will use 10 ions per turn. The number of ions per turn has important biophysical implications: conservation of energy requires that it is proportional to the maximum torque and force that the motor can exert. Torque is an important parameter for the function of any motor – it determines what the motor can drive, and, in the case of dissipative loads, how fast it can be driven. For example, in the case of MotAB, the maximum torque that can be generated determines the ability of the BFM to rotate flagella in viscous media. The maximum average torque τ produced by a rotary motor depends on the number of quanta of “fuel” it uses per revolution $N_{r}$ and on the free energy ΔG released per quantum (one ATP molecule hydrolyzed or one ion transited), as $\tau =\frac{N_{r}\;\Delta G}{2\pi }.$ The stoichiometry of F_O_F_1_ ATP synthase constitutes a good illustration of the importance of this relation. Each revolution is coupled to the transit of *n* protons through F_O_ (*n = 10* in *E. coli* (Jiang et al., 2001), but *n* varies widely among species) and the synthesis or hydrolysis of 3 ATP molecules in F_1_. For the complex to convert the free energy of protons to that of the synthesis of ATP with high efficiency, the energy $\tau_{O}\delta \theta$ released by the proton flux when F_O_ rotates through an angle δθ should be minimally larger than the energy $\tau_{1}\delta \theta$ absorbed by ATP synthesis in F_1_. Thus, $\tau_{O}≳\tau_{1}$ , and therefore $\frac{\Delta G_{ATP}}{\Delta G_{H^{+}}}≲\frac{n}{3}.$ $\Delta G_{ATP}$ depends on the pH and concentrations of ATP, magnesium, ADP, and phosphate. For typical metabolite concentrations in *E. coli* (Tran and Unden, 1998; Bennett et al., 2009), this corresponds to $\sim 20\;k_{B}T=50\;kJ/mol$ . On the other hand, the PMF across the inner membrane of *E. coli* is typically ~170 mV (Cheuk and Meier, 2021). Thus, the free energy per proton $\Delta G_{H^{+}}=e\Delta V≃$ 16 kJ/mol, and $\frac{\Delta G_{ATP}}{\Delta G_{H^{+}}}≃3.1$ . This is slightly less than 10/3, exactly as expected. Increasing the stoichiometry of the c-ring from 11 to 12 allows the ATP synthase to work at a lower ion motive force (Pogoryelov et al., 2012), as predicted by the energetic argument presented here. A meta-study showed that species with a higher c-ring stoichiometry, like *Bacillus pseudofirmus* (*n* = 13), have a lower PMF (~150 mV), whereas ATP synthases with a lower c-ring stoichiometry, like those of *Bos taurus* mitochondria (*n* = 8), have a higher PMF (~210 mV). The lack of precise data regarding PMF and $\Delta G_{ATP}\;$ in all these species limits the rigor of this comparison. However, these considerations suggest that the stoichiometry of the F_O_F_1_ ATP synthase has evolved to favor efficient energetic coupling between PMF and ATP synthesis, demonstrating that torque and structural symmetry are intimately related.

### Comparison of expected maximum torque and the thermodynamics of coiled coils

In *E. coli*, both 5:2 rotary motors and F_O_ use *n* = 10 protons per revolution and generate a maximum torque of ~40 pN.nm. Is this value special in any way, or is it just a coincidence? Both the BFM and ATP synthase require internal “anchoring” of components relative to each other, to prevent free rotation that would dissipate the proton energy as heat. In the BFM, the MotB dimers play this role by anchoring stator units into the peptidoglycan (Kojima et al., 2018). In *E. coli* F_O_F_1_ ATP synthase, the “second stalk” dimer of b-subunits prevents rotation of the a-subunit of F_O_ relative to the cap of F_1_ (Dmitriev et al., 1999), ensuring that F_O_ rotation drives the conformational changes within F_1_ that synthesize ATP. Both of these anchoring dimers are α-helical coiled coils (del Rizzo et al., 2006; Wise and Vogel, 2008; Andrews et al., 2017; Sobti et al., 2020). There is some disagreement on the handedness of the coiled coils in the *E. coli* F_O_F_1_ ATP synthase, and recent cryoEM studies of the F_O_F_1_ ATP synthase of *Saccharomyces cerevisiae* suggest that the handedness can change upon energization and under stress (Guo and Rubinstein, 2022). On the other hand, isothermal calorimetric thermodynamic measurements of coiled coiling (Jelesarov and Bosshard, 1996; Worrall and Mason, 2011) between two α-helices found consistent values for the standard free energy change $\Delta G_{coiling}^{{}^{\circ}}$ between uncoiled and coiled states, which is about 9 kcal/mol for protein sequences that are four heptads long (a heptad corresponds to seven amino acids, two turns of an α-helix). Since one turn of a coiled-coil corresponds to 80–120 residues (Seo and Cohen, 1993; Lupas and Gruber, 2005; Sharp et al., 2012), four heptads correspond to a torsion angle φ of 1.45–2.2 rad. Thus, the characteristic uncoiling torque associated with the measured $\Delta G_{coiling}^{{}^{\circ}}$ is $\tau =\frac{\Delta G_{coiling}^{{}^{\circ}}}{\varphi }=30-40\;pN.nm$ , close to the predicted maximum torque generated by MotAB and F_O_. These thermodynamic considerations, and the observation that 10-fold periodicity is shared by F_O_ and presumably all 5:2 rotary motors, lead us to speculate that the maximum periodicity of these minimal motors could be limited by the maximum torque that can be withstood by the anchoring coiled coils. These quantities are rough estimates. The true dynamics and energetics of anchoring will be much more complex. In the case of F_O_F_1_ ATP synthases for example, the b-subunits are shifted from the axis of rotation and thus will be subject to a mixture of torque, tension, and bending stress. Single-molecule studies of those dimers and their ability to withstand and react to the applied torque, following the model of the experiments that have been performed on DNA (Strick et al., 1996), could test this hypothesis.

Finally, we note that in the case of the hypothetical TolQR and ExbBD *rotate-and-pull* mechanism discussed in this review, the most important parameter is not the torque but rather the force applied to TolA and TonB, respectively. The force transmitted to a protein attached to the outer side of the complex is directly proportional to the motor torque and inversely proportional to the outer radius of the complex. Exerting a high enough force on the TolB or TonB-box motifs thus requires that a rotary motor combines a large number of protons per turn (*n = a x b*) with a small outer radius. 5:2 may be the best symmetry to meet this requirement.

## Author contributions

MR proposed models explaining how functions of known 5:2 motors could accommodate rotation, composed the corresponding parts of the section “Functions as components of larger systems,” and composed the section “General considerations of rotary motor symmetry.” RK composed the section “Phylogenetics.” RK and NT composed the section “Structures of ion-driven 5:2 rotary motors.” RB composed the Introduction, the “MotAB” part of the section “Functions as components of larger systems,” and the Section “A model for the rotary mechanism” and assembled and edited the manuscript. All authors contributed to the revision of the manuscript.

## Funding

RB and MR are supported by UK Research and Innovation (UKRI, Engineering, and Physical Sciences Research Council) grant EP/S036660/1. The Novo Nordisk Foundation Center for Protein Research is supported financially by the Novo Nordisk Foundation (grant NNF14CC0001). This work was also supported by an NNF Hallas-Møller Emerging Investigator grant (NNF17OC0031006) to NT is a member of the Integrative Structural Biology Cluster (ISBUC) at the University of Copenhagen.

## Conflict of interest

The authors declare that the research was conducted in the absence of any commercial or financial relationships that could be construed as a potential conflict of interest.

## Publisher’s note

All claims expressed in this article are solely those of the authors and do not necessarily represent those of their affiliated organizations, or those of the publisher, the editors and the reviewers. Any product that may be evaluated in this article, or claim that may be made by its manufacturer, is not guaranteed or endorsed by the publisher.
