# Supplementary figures and images for: A new class of biological ion-driven rotary molecular motors with 5:2 symmetry
Source: Front Microbiol. 2022 Aug 5;13:948383. doi: 10.3389/fmicb.2022.948383 (PMC9389320; doi:10.3389/fmicb.2022.948383)

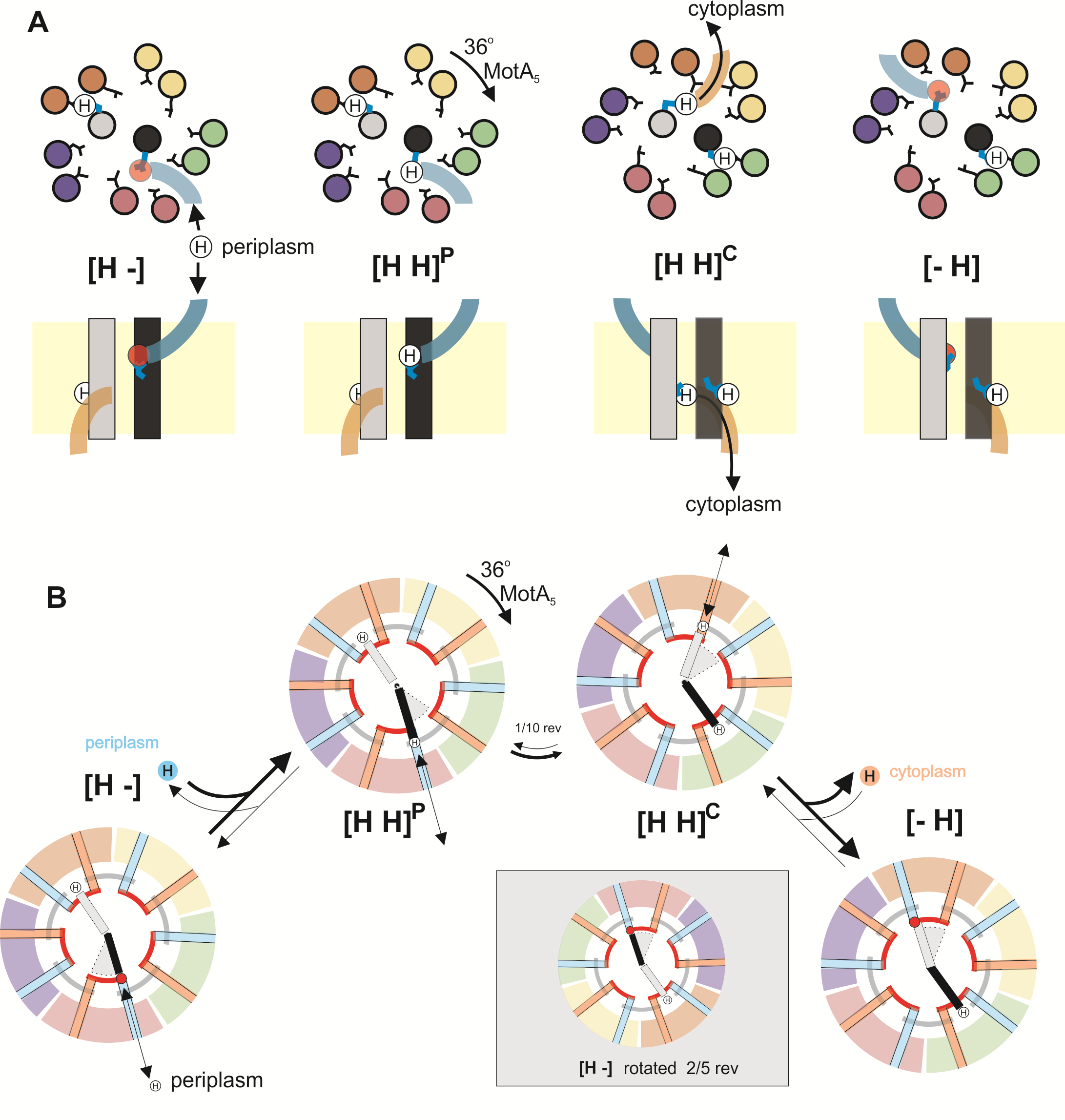

Supplement: Supplementary file 1 [file Image_1.tif]
